# Patients' experiences across the trajectory of atrial fibrillation: A qualitative systematic review

**DOI:** 10.1111/hex.13451

**Published:** 2022-02-17

**Authors:** Jie Wang, Shenxinyu Liu, Zhipeng Bao, Min Gao, Yuanyuan Peng, Yangxi Huang, Tianxi Yu, Lin Wang, Guozhen Sun

**Affiliations:** ^1^ School of Nursing, Nanjing Medical University Nanjing Jiangsu China; ^2^ Department of Cardiology The First Affiliated Hospital of Nanjing Medical University Nanjing Jiangsu China; ^3^ School of Nursing, Sanda University Shanghai China

**Keywords:** atrial fibrillation, coping strategies, demands, diagnosis and treatment, qualitative synthesis

## Abstract

**Aim:**

This study aimed to synthesize qualitative evidence on experiences of patients with atrial fibrillation (AF) during the course of diagnosis and treatment. We addressed three main questions: (a) What were the experiences of patients with AF during the course of diagnosis and treatment? (b) How did they respond to and cope with the disease? (c) What were the requirements during disease management?

**Design:**

In this study, qualitative evidence synthesis was performed using the Thomas and Harden method.

**Data Sources:**

Electronic databases, including PubMed, the Cochrane Library, Embase, Web of Science, Cumulative Index to Nursing and Allied Health Literature, the China Biomedical Database, the WanFang Database, Chinese National Knowledge Infrastructure and VIP, were searched. The databases were searched from inception to August 2021.

**Review Methods:**

Two researchers independently selected studies using qualitative assessment and review instruments for quality evaluation and thematic synthesis for the data analysis.

**Results:**

A total of 2627 studies were identified in the initial search and 15 studies were included. Five analytical themes were generated: ‘Diagnosing AF’; ‘The impact of AF on the patients’; ‘Self‐reorientation in the therapeutic process’; ‘Living with AF and QoL’; and ‘External support to facilitate coping strategies.’

**Conclusions:**

Our findings point out unique experiences of patients across the trajectory of AF related to delayed diagnosis, feelings of nonsupport, disappointment of repeated treatment failure and multiple distress associated with unpredictable symptoms. Future research and clinical practice are expected to improve the quality of medical diagnosis and treatment, optimize administrative strategy and provide diverse health support for patients with AF.

**Impact:**

Understanding the experiences and needs of patients with AF in the entire disease process will inform future clinical practice in AF integrated management, which would be helpful in improving the professionalism and confidence of healthcare providers. In addition, our findings have implications for improving the effectiveness of AF diagnostic and treatment services.

**Patient or Public Contribution:**

This paper presents a review of previous studies and did not involve patients or the public.

## 1 INTRODUCTION

Atrial fibrillation (AF) is the most common persistent type of cardiac arrhythmia occurring clinically, and can lead to serious complications. According to the European Society of Cardiology (2017), AF is defined as supraventricular tachyarrhythmia with uncoordinated atrial electrical activation and consequent ineffective atrial contraction.^1^ Globally, the average AF prevalence ranges from 2% to 4%. In China, epidemiological data show that the prevalence of AF is approximately 0.7% in the overall population; this number increases with age, and a prevalence of 7.5% is observed among adults over 80 years of age.^2^ AF‐related complications include thromboembolism and heart failure in severe cases.^3^, ^4^ As the disease progresses, patients with AF experience various heart symptoms and physical discomfort.^5^ Moreover, AF also affects psychological well‐being, social connectedness and quality of life (QoL).^6^

In recent years, numerous qualitative studies have explored the feelings and thoughts of AF patients at symptom onset, while seeking treatment and during recovery after the right treatment. However, each study has its own unique limitations, making it difficult to identify and summarize the general problems and challenges affecting the provision of high‐quality care for AF patients. Qualitative studies addressing this topic have not yet been synthesized. The aggregation of findings from qualitative research is gaining importance for evidence‐based healthcare. Therefore, the aim of this qualitative systematic review was to identify and analyse the perceptions and feelings of AF patients to provide a reference for developing family‐ and community‐based nursing strategies for meeting the care needs of AF patients. To do so, we critically appraised and synthesized existing qualitative research exploring the experiences and needs of AF patients in the course of diagnosis and treatment.

## 2 THE REVIEW

### 2.1 Aim

The aim of this review was to synthesize qualitative evidence on the experiences of patients with AF during the course of diagnosis and treatment. We addressed three main questions: (a) What were the experiences of patients with AF during the course of diagnosis and treatment? (b) How did they respond to and cope with the disease? (c) What were the requirements during disease management?

### 2.2 Design

A systematic review and thematic synthesis of qualitative studies were conducted. This review was undertaken according to the Enhanced Transparency of Reporting the Synthesis of Qualitative Research framework (ENTREQ).^7^ This framework provides guidance on the synthesis of qualitative research and ensures the quality and rigour of the study.

### 2.3 Search methods

A comprehensive search of the literature published from inception to August 2021 was conducted in the following electronic databases: PubMed, Cochrane Library, Embase, Web of Science, Cumulative Index to Nursing and Allied Health Literature, the China Biomedical Database, the WanFang Database, Chinese National Knowledge Infrastructure and  the Chinese Scientific Journals Database (VIP). The search terms were grouped into three blocks (Table 1).

**Table 1:** Search terms

Search block	Search items
Population	‘Atrial Fibrillation’ OR ‘Auricular Fibrillation’ OR ‘atrium fibrillation’ OR ‘Paroxysmal atrial fibrillation’ OR ‘Persistent atrial fibrillation’ OR ‘PAF’ OR ‘PeAF’ OR ‘AF’
Experience	‘illness experience’ OR ‘experience’ OR ‘feeling*’ OR ‘symptom*’ OR ‘need*’ OR ‘demand’ OR ‘perception’ OR ‘thought*’ OR ‘preference’ OR ‘attitude’
Study design	‘qualitative research’ OR ‘qualitative study’ OR ‘grounded theory’ OR ‘focus group’ OR ‘participant observation’ OR ‘phenomenology’ OR ‘action research’

### 2.4 Criteria for inclusion and exclusion

The following eligibility criteria were applied:

1. Articles investigating the experiences, values and expectations of patients aged 18 years and older who are diagnosed with AF, with no limitations on the type of AF.
2. English or Chinese articles published from inception to August 2021.
3. Articles focusing on contexts including symptom onset, seeking medical diagnosis and treatment and self‐management after treatment (covering the entire process of diagnosing and treating disease).

Studies were excluded if they fulfilled the following criteria: Articles only explored the experiences related to anticoagulation therapy as it was considered that this exclusion did not detract from the study's efficacy; studies that were quantitative, mixed studies or reviews were excluded.

### 2.5 Search outcomes

**Figure 1:**
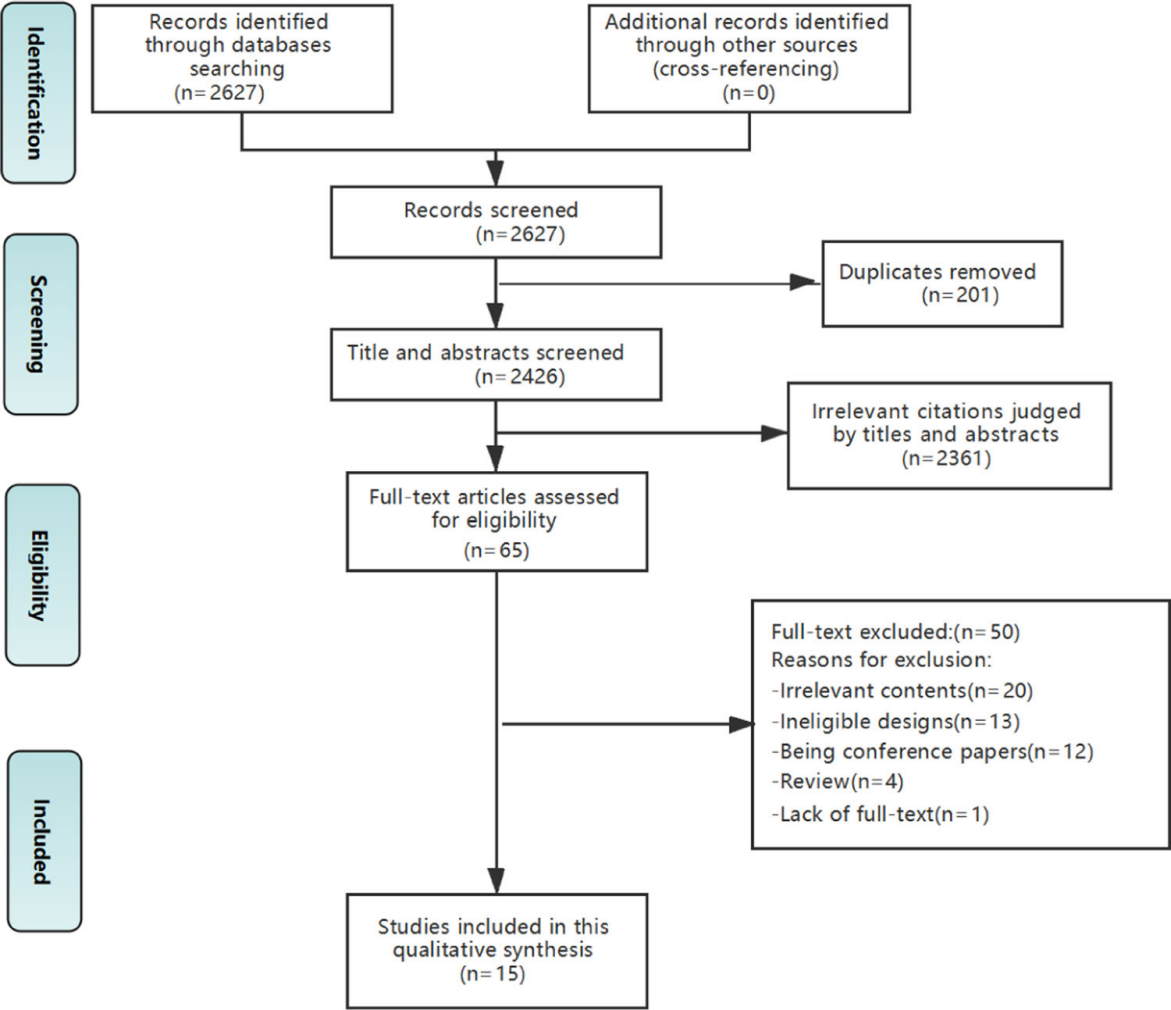
Flow chart of study selection

According to the search strategy, a total of 2627 studies were extracted from the databases. After the removal of duplicates, the researchers reviewed 2426 titles and abstracts and collected 50 full‐paper studies for further review. In total, 15 qualitative studies fulfilled the inclusion criteria and were included, of which 13 were in English and two were in Chinese. The literature screening and selection process is shown in Figure 1.

### 2.6 Quality appraisal

The quality of all the included studies was assessed using the Critical Appraisal Skills Programme (CASP) qualitative appraisal instrument; the rigour and credibility of the studies were assessed. Two authors independently rated the quality of the studies and discussed incongruity issues until a consensus was reached. Thirteen studies fulfilled nine or more of the 12 quality criteria, whereas the remaining two fulfilled 7 criteria (Table 2). The three domains that form Consolidates Criteria for Reporting Qualitative Research (COREQ) are as follows: the research team and reflexivity, study design and analysis and findings. Researchers used the COREQ checklist to further assess the transparency of reporting. Two studies were not assessed because their methodological designs did not satisfy the above‐mentioned criteria (Table 3). Finally, we performed sensitivity analyses to assess the possible effect of study quality on the review's findings as well as the contributions of the study to the final synthesis.

**Table 2:** Quality assessment based on the CASP Qualitative Research Checklist (2018)

Selected studies	CASP checklist criteria										Meeting quality criteria	Quality grade
	1	2	3	4	5	6	7	8	9	10		
Altiok et al. (2015)	Y	Y	Y	Y	Y	Y	Y	Y	Y	Y	10	A
Bergtun et al. (2019)	Y	Y	Y	Y	Y	U	Y	Y	Y	Y	9	B
Deaton et al. (2003)	Y	Y	Y	U	Y	U	U	Y	Y	Y	7	B
McCabe et al. (2011)	Y	Y	Y	Y	Y	Y	Y	Y	Y	Y	10	A
McCabe et al. (2015)	Y	Y	Y	Y	Y	Y	Y	Y	Y	Y	10	A
Nørgaard et al. (2015)	Y	Y	Y	Y	Y	Y	Y	Y	Y	Y	10	A
Redman et al. (2017)	Y	Y	Y	U	Y	Y	Y	Y	Y	Y	9	B
Rush et al. (2015)	Y	Y	Y	Y	Y	Y	Y	Y	Y	Y	10	A
Salmasi et al. (2018)	Y	Y	Y	Y	Y	U	Y	Y	Y	Y	9	B
Taylor et al. (2017)	Y	Y	Y	Y	Y	Y	Y	Y	Y	Y	10	A
Thrysoee et al. (2018)	Y	Y	Y	U	Y	Y	Y	Y	Y	Y	9	B
Wilson et al. (2020)	Y	Y	Y	Y	Y	Y	Y	Y	Y	Y	10	A
Shen et al. (2020)	Y	Y	Y	Y	Y	U	U	U	Y	Y	7	B
Li et al. (2014)	Y	Y	Y	Y	Y	Y	Y	Y	Y	Y	10	A
McCabe et al. (2020)	Y	Y	Y	Y	Y	U	Y	Y	Y	Y	9	B

*Note*: CASP criteria for qualitative studies: (1) Was there a clear statement of the aims of the research? (2) Is a qualitative methodology appropriate? (3) Was the research design appropriate to address the aims of the research? (4) Was the recruitment strategy appropriate to the aims of the research? (5) Was the data collected in a way that addressed the research issue? (6) Has the relationship between the researcher and participants been adequately considered? (7) Have ethical issues been taken into consideration? (8) Was the data analysis sufficiently rigorous? (9) Is there a clear statement of the findings? (10) How valuable is the research?

Abbreviations: CASP, Critical Appraisal Skills Programme; N, no; U, unclear/cannot tell; Y, yes.

*Source*: https://casp-uk.net/wp-content/uploads/2018/03/CASP-Qualitative-Checklist-2018_fillable_form.pdf

**Table 3:** Application and scoring of papers included in review (two studies not suitable for the checklist were excluded)

	Research team and reflexivity score/14								Study design score/24															Analysis and findings score/12								
	1. Interviewer/facilitator	2. Credentials	3. Occupation	4. Gender	5. Experience and training	6. Relationship established	7. Participant knowledge of interviewer	8. Interviewer characteristics	9. Methodological orientation and theory	10. Sampling	11. Method of approach	12. Sample size	13. Nonparticipation	14. Setting of data collection	15. Presence of non‐participants	16. Description of sample	17. Interview guide	18. Re[eat interviews	19. Audio/visual recordings	20. Field notes	21. Duration	22. Data saturation	23. Transcripts returned	24. Number of data codes	25. Description of coding tree	26. Derivation of themes	27. Software	28. Participant checking reporting	29. Quotations presented	30. Data and findings consistent	31. Clarity of major themes	32. Clarity of minor themes
Altiok et al. (2015)	N	Y	N	N	N	N	Y	N	Y	Y	Y	Y	Y	Y	N	Y	Y	N	Y	Y	Y	Y	N	N	Y	Y	Y	Y	Y	Y	Y	Y
Bergtun et al. (2019)	Y	Y	Y	N	N	N	Y	N	Y	Y	Y	Y	Y	Y	N	Y	Y	N	Y	N	Y	Y	N	N	Y	Y	N	N	Y	Y	Y	Y
Deaton et al. (2003)	N	Y	N	N	N	N	Y	N	Y	Y	N	Y	N	Y	N	Y	N	N	Y	N	N	N	N	N	Y	Y	Y	N	Y	Y	Y	Y
McCabe et al. (2011)	N	Y	Y	N	N	N	Y	N	Y	Y	Y	Y	N	Y	N	Y	Y	N	Y	Y	Y	Y	Y	N	Y	Y	N	Y	N	Y	Y	Y
McCabe et al. (2015)	Y	Y	N	N	N	Y	Y	N	Y	Y	Y	Y	N	Y	Y	Y	Y	N	Y	Y	Y	Y	N	N	Y	Y	Y	N	N	Y	Y	Y
Nørgaard et al. (2015)	Y	N	N	N	Y	Y	Y	N	Y	Y	Y	Y	Y	Y	Y	Y	Y	N	Y	N	Y	Y	N	N	Y	Y	N	N	Y	Y	Y	Y
Rush et al. (2015)	Y	N	Y	N	Y	Y	Y	N	Y	Y	Y	Y	Y	Y	N	Y	Y	N	Y	N	Y	Y	N	N	Y	Y	Y	N	Y	Y	Y	Y
Salmasi et al. (2018)	Y	Y	N	N	N	N	Y	N	Y	Y	Y	Y	N	Y	Y	Y	Y	N	Y	Y	Y	Y	N	N	Y	Y	Y	N	N	Y	Y	Y
Taylor et al. (2017)	Y	N	N	N	Y	N	Y	N	Y	Y	Y	Y	N	Y	N	Y	Y	N	Y	Y	Y	Y	N	N	Y	Y	Y	Y	Y	Y	Y	Y
Wilson et al. (2020)	Y	Y	N	N	Y	N	Y	N	Y	Y	Y	Y	N	Y	N	Y	Y	N	Y	Y	Y	Y	N	N	Y	Y	Y	N	Y	Y	Y	Y
Shen et al. (2020)	Y	Y	Y	N	Y	N	Y	N	Y	Y	Y	Y	N	Y	N	Y	Y	N	Y	Y	Y	Y	N	N	Y	Y	N	N	Y	Y	Y	Y
Li et al. (2014)	Y	Y	Y	N	Y	N	Y	N	Y	Y	N	Y	Y	Y	Y	Y	Y	N	Y	Y	Y	Y	Y	N	Y	Y	Y	N	Y	Y	Y	Y
McCabe et al. (2020)	Y	Y	Y	N	Y	N	N	N	Y	Y	Y	Y	N	Y	Y	Y	Y	N	Y	N	Y	Y	N	N	Y	Y	Y	N	N	Y	Y	Y

*Note*: Two studies not suitable for the checklist were excluded.

Consolidated criteria for reporting qualitative studies (COREQ): 32‐item checklist: (1) Which author(s) conducted the interview or focus group? (2) What were the researcher's credentials? e.g., PhD, MD, and so forth. (3) What was their occupation at the time of the study? (4) Was the researcher male or female? (5) What experience or training did the researcher have? (6) Was a relationship established before study commencement? (7) What did the participants know about the researcher? e.g., personal goals, reasons for doing the research, and so forth. (8) What characteristics were reported about the interviewer/facilitator? e.g., bias, assumptions, reasons and interests in the research topic. (9) What methodological orientation was stated to underpin the study? e.g., grounded theory, discourse analysis, ethnography, phenomenology, content analysis, and so forth. (10) How were participants selected? e.g., purposive, convenience, consecutive, snowball. (11) How were participants approached? e.g., face‐to‐face, telephone, mail, email. (12) How many participants were in the study? (13) How many people refused to participate or dropped out? Reasons? (14) Where was the data collected? e.g., home, clinic, workplace, and so forth. (15) Was anyone else present besides the participants and researchers? (16) What are the important characteristics of the sample? e.g., demographic data, and so forth. (17) Were questions, prompts, guides provided by the authors? Was it pilot tested? (18) Were repeat interviews carried out? If yes, how many? (19) Did the research use audio or visual recording to collect the data? (20) Were field notes made during and/or after the interview or focus group? (21) What was the duration of the interviews or focus groups? (22) Was data saturation discussed? (23) Were transcripts returned to participants for comment and/or correction? (24) How many data coders coded the data? (25) Did authors provide a description of the coding tree? (26) Were themes identified in advance or derived from the data? (27) What software, if applicable, was used to manage data? (28) Did participants provide feedback on the findings? (29) Were participant quotations presented to illustrate the themes/findings? Was each quotation identified? e.g., participant number. (30) Was there consistency between the data presented and the findings?  (31) Were major themes clearly presented in the findings? (32) Is there a description of diverse cases or discussion of minor themes?

Abbreviations: N, no; Y, yes.

### 2.7 Data abstraction

The details of each study were extracted and complied in an Excel file (see Table 4). The main contexts of data extraction included authorship, year of publication and country, methodological design and data collection, sample and sample size, research question and findings relevant to the review.

**Table 4:** Characteristics of the studies included in the systematic review

First author (year)	Country of origin	Methodological design and data collection	Sample and sample size	Research question	Findings relevant to the review
Altiok (2015)	Turkey	Phenomenological research/semi‐structured interviews	32 Adults patients diagnosed with AF > 6 months	To investigate their perspectives and coping behaviours towards their condition	1. Mental status regarding the disease
					2. Social status regarding the disease
					3. Physical condition regarding the disease
					4. Disease management and coping with disease
Bergtun (2019)	Norway	Deductive qualitative research/semi‐unstructured, interviews	19 Patients with AF (11 males and 8 females)	To describe patients' experiences from a holistic perspective 1–6 months after AF ablation	1. Having unexpected complications with a slower recovery
					2. Discovering one's own self‐management strategies when lacking information and insufficient follow‐up
					3. Managing resentment through different coping strategies while emotional reactions depended on feeling better or worse
					4. Failing to receive full understanding and support from close ones, with social consequences when the biophysical level did not return to normal
					5. Gradually adopting new life perspectives with a hope for a better future, despite having unmet expectations and uncertainty, leading to discovery of existential matters
Deaton (2003)	America	Descriptive qualitative research/semi‐structured interviews	11 ICD‐AT patients (3 females and 8 males)	To describe experience of patients living with symptomatic, drug‐refractory AF and acceptance of treatment with ICD‐AT	1. Pre‐ICD‐AT implant themes: process of seeking a diagnosis and treatment plan
					2. Decision‐making and device implantation: the end of the road
					3. Post‐ICD‐AT themes: living with the ICD‐AF
McCabe (2011)	America	Descriptive qualitative research/open‐ended interviews	15 Patients undergoing treatment with an antiarrhythmic drug or scheduled for ablation therapy for AF (7 females and 8 males)	To describe experience of living with recurrent symptomatic AF from patients' perspectives	1. Finding the meaning of symptoms
					2. Feeling uninformed and unsupported
					3. Turning points
					4. Steering clear of AF
					5. Managing unpredictable and function‐limiting symptoms
					6. Emotional distress
					7. Accommodation to AF tempered with hope for a cure
McCabe (2015)	America	Descriptive qualitative research/open‐ended interviews	41 Patients with AF (20 females and 21 males)	To describe patients' experiences from symptom onset to initial treatment for AF	1. Misinterpreting symptoms
					2. Discovering the meaning of atrial fibrillation
					3. Facing fears, uncertainty and moving to acceptance
					4. Receiving validation and reassurance
Nørgaard (2015)	Denmark	Qualitative research/semi‐structured interviews	14 Patients receiving visualization intervention during ablation of AF (3 females and 11 males)	To investigate patients' experiences with visualization in relation to pain and anxiety during an intervention consisting of visualization, when undergoing ablation of AF	1. Approach to visualization
					2. Strategies of managing pain
					3. Strategies of managing anxiety
					4. Benefits of visualization
Redman (2017)	Canada	Interpretive descriptive research/qualitative, nonparticipant, observational approach	103 Unique user names participated in the discussion; 181 threads were analysed	To determine the content and dialogue on an online message board for AF with the purpose of elucidating information and support needs from patient perspectives	1. Sharing experiences and values
					2. Searching for sense
					3. Managing the complexities of information
					4. Acting as a wise consumer
Rush (2015)	Canada	Descriptive qualitative research/semi‐structured interviews	16 Patients with AF (13 males and 3 females)	To explore the stressors and coping strategies of older adults with persistent AF before and after direct current cardioversion	1. Pre‐ and postprocedure Stressors: AF symptoms and impact, healthcare and treatment, non‐AF stressors
					2. Self‐management and coping strategies: emotion‐focused coping, problem‐focused coping
Salmasi (2018)	Canada	Descriptive qualitative research/semi‐structured interviews	10 Patients with AF (8 males and 2 females)	To gather insights into AF patients' education needs from patient and clinician viewpoints	1. Emotional appraisal of the disease
					2. Information‐seeking behaviour
					3. Knowledge gaps
					4. Education preferences
Taylor (2017)	England	Grounded theory/semi‐structured and open‐ended interviews	30 Patients with persistent AF (19 males and 11 females)	To examine patients' illness and treatment beliefs and ways of coping with AF symptoms	1. Unpredictability and uncertainty of AF and symptoms
					2. Coping with symptoms
					3. Concerns and expectations about treatment
Thrysoee (2018)	Denmark	Ethnographic research/participant observation and semi‐structured individual interviews	14 Patients newly diagnosed with AF (7 males and 7 females)	To gain knowledge of patients' experiences of the consultation processes at a multidisciplinary AF outpatient clinic in a university hospital in Denmark	1. Uncertainty about AF before first consultation
					2. Focus on the medical aspects of AF
					3. AF is not a fatal disease
					4. Professionalism and competence in the care of AF
					5. Visiting the AF‐clinic—an overwhelming experience
Wilson (2020)	Canada	Interpretive descriptive research/semi‐structured telephone interviews	26 participants received an AF diagnosis within the 12 months before interviews (13 males and 13 females)	To explore the symptom experiences of patients receiving an early diagnosis of <48 h and a late diagnosis of ≥48 h after symptom awareness	1. Symptom perception: symptom characteristic, imperceptible noticing, commanding attention, rest and activity
					2. Symptom evaluation: overall lack of concern, self‐derived theorizing, finding support for their theories, retheorizing after self‐derived theorizing, theory disruption
					3. Symptom response: nontreatment, self‐treatment, healthcare seeking
Shen (2020)	China	Qualitative research/semi‐structured interviews	15 Patients undergoing radiofrequency ablation of AF (9 females and 6 males)	To understand the self‐experience and nursing needs of patients with AF after radiofrequency ablation	1. Relief of symptoms
					2. Worrying about the prognosis
					3. Meeting the demands of in‐patients
					4. Strong demand for transitional care
Li (2014)	China	Phenomenological research/semi‐structured depth interviews	10 AF patients undergoing radio‐ frequency ablation under the introduction of EnSite‐NavX (4 females and 6 males)	To explore the psychological experience, coping styles and the internal needs of the patients with AF during the course of radio‐ frequency ablation under the introduction of EnSite‐NavX	1. Various complicated emotional responses
					2. Various body discomforts
					3. Adopting various ways to respond to the discomforts of the body and physiology
					4. Requirements of the medical workers' technical operation and service attitude
McCabe (2020)	America	Qualitative research/semi‐structured interviews	25 Patients diagnosed with AF <18 months (8 females and 17 males)	To explore patients' values concerning the content of initial AF education, describe how providers delivered education and identify patients' preferences for approaches to education	1. Important to know
					2. Recollections of the how and what of education
					3. Preferences for educational resources

Abbreviation: AF, atrial fibrillation; ICD‐AT, the implantable cardioverter defibrillator with atrial therapies.

### 2.8 Data synthesis

Data were analysed by two researchers (W. J. and L. S.) using a three‐stage thematic synthesis method.^8^ First, QSR NVivo software was used to code the text line by line according to the key findings of the primary studies. Then, the researchers checked the similarities and differences across the codes to obtain descriptive themes. Finally, analytical themes were developed by repeatedly reviewing and analysing the descriptive themes.

## 3 FINDINGS

### 3.1 Characteristics of the included studies

**Figure 2:**
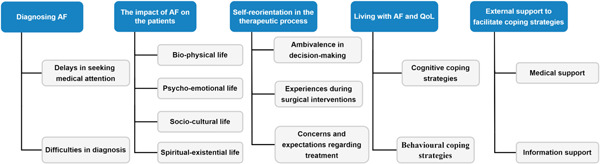
Synthesized themes and subthemes

The 15 qualitative studies represented 381 participants with AF originating from eight countries (America, Canada, China, Denmark, England, Norway, Sweden and Turkey). The study characteristics are summarized in Table 4. The data of interest were synthesized into five analytical themes with 13 descriptive subthemes (Figure 2). As the sensitivity analyses show, the findings synthesized did not contradict each other, and no matter which article was removed, the results would be the same. A summary of each study's contribution to the synthesis is presented in Table 5. Both descriptive and analytical findings were reported as detailed below.

**Table 5:** Sensitivity analysis

Themes	Altiok (2015)	Bergtun (2019)	Deaton (2003)	McCabe (2011)	McCabe (2015)	Nørgaard (2015)	Redman (2017)	Rush (2015)	Salmasi (2018)	Taylor (2017)	Thrysoee (2018)	Wilson (2020)	Shen (2020)	Li (2014)	McCabe (2020)
Delays in seeking medical attention					√		√				√	√			
Difficulties in diagnosis			√	√	√					√	√	√			
Biophysical life		√	√					√	√	√					
Psycho‐emotional life	√	√	√	√			√	√		√	√		√		
Sociocultural life	√	√	√				√					√			
Spiritual‐existential life	√	√	√				√	√				√			
Ambivalence in decision‐making		√	√					√		√	√		√		√
Experiences during surgical interventions						√								√	
Concerns and expectations regarding treatment	√	√	√	√				√	√	√			√		
Cognitive coping strategies	√	√		√	√			√		√	√	√			
Behavioural coping strategies	√	√		√			√	√		√		√			
Medical support	√				√	√		√	√		√			√	
Information support	√		√	√	√		√	√	√	√	√	√			√

### 3.2 Diagnosing AF

The studies described several obstacles to establishing a definitive diagnosis of AF. These included delays in seeking medical attention and difficulties in diagnosing new‐onset AF due to multiple reasons. These two aspects are discussed below.

#### 3.2.1 Delays in seeking medical attention

Symptom perception varied significantly among patients in the early stages of AF; this is because of differences in factors including the duration of symptoms, frequency of attacks, form of presentation and perceived susceptibility, which influenced attitudes towards seeking medical care. Due to a lack of knowledge about AF, some patients chose to attribute the symptoms to non‐disease causes such as senescence and stress, even though the symptoms included dyspnoea, excessive fatigue and other AF‐related symptoms. These misattributions may cause patients to delay their visit to the doctor. Such patients chose to continue to endure the symptoms or used a wait‐and‐see strategy. More importantly, AF episodes may be asymptomatic in some cases. Patients with silent AF usually neglect the importance of timely medical treatment.^9^, ^10^, ^11^, ^12^, ^13^

Hoping this was all just stress related. So, I didn't necessarily. I didn't make an appointment right away with my family doctor. I was hoping it'd go away.^9^

#### 3.2.2 Difficulties in diagnosis

As the symptoms gradually aggravated, patients ultimately chose to seek medical help. However, there were other obstacles to obtaining a clear diagnosis of AF. On the one hand, early AF was mostly paroxysmal, and was difficult to capture. Many patients experienced sudden chest discomfort, and this sensation receded quickly. Such an episode would not likely register as an indicator of disease even at a fully equipped hospital; this may lead to the omission of the potential risk of AF. On the other hand, it was reported that doctors often did not pay enough attention to patients' symptom feedback and interpreted the patient symptoms to be a result of anxiety, stress and female menopause. This directly influenced the ability of the patients to cope with the symptoms. The dramatic contrast between symptom experience and physician judgement was questioned by a few patients and made them even more eager to find the true cause of the symptoms. A definitive diagnosis of AF brings relief to the patients and provides an explanation for the patient's symptoms to others.^10^, ^12^, ^13^, ^14^, ^15^, ^16^

I told them my heart was racing—I feel a little weak—they hooked me up and ran the EKG—and at that point I told them OK—I need to get out of here—please send that up to the clinic. They looked at me like I was nuts.^15^ Well, I was very happy to have a label, finally, and find out what it really was.^10^

### 3.3 The impact of AF on the patients

The impact of AF on people was multifaceted. The study patients reported a wide range of physical symptoms and psychological, social and existential concerns.

#### 3.3.1 Biophysical life

A perception of decreased physical functioning was the most common problem reported by the patients. It is well known that physical fitness and level of daily activities are highly interrelated. With disease progression, patients' health states show degeneration, and they are no longer able to perform activities such as walking, cooking, shopping and housework. Some patients even lose their self‐care abilities.^14^, ^16^, ^17^, ^18^, ^19^, ^20^

I love shopping, hanging around in shopping malls or markets, or spending money, but I cannot go to these places myself.^17^

#### 3.3.2 Psycho‐emotional life

The limitations of the illness also affected the patients' psycho‐emotional profile. Negative emotions were experienced by patients; this encouraged the patients to make effective use of internal and external resources to avoid symptoms and gain peace of mind. Nevertheless, when unpredictable symptoms occurred, patients were observed to engage in ruminative thinking, to the detriment of their peace of mind.^11^, ^12^, ^14^, ^15^, ^16^, ^17^, ^18^, ^19^, ^21^

I've had episodes lasting for hours. Then, I'm incredibly anxious, almost in agony. Don't know… you can't do anything; you feel helpless. You sit and wait, ‘am I dying now?’ So, it's almost torture—a torture chamber.^18^

#### 3.3.3 Sociocultural life

A decrease in social activities and worsening financial situations both accelerated the decline in the QoL of patients. The inability to engage in hobbies forced some patients to choose other options of entertainment or to forego entertaining activities completely, causing their social circles to shrink further. In addition, the high costs associated with treating this disorder aggravated the medical financial burden of households.^11^, ^13^, ^14^, ^17^, ^18^

#### 3.3.4 Spiritual‐existential life

To a certain extent, the shift in patients' roles or identities undermined their sense of self‐worth and dignity. Patients were labelled as sick persons because of their decreased functioning. Due to lack of energy, they were unable to fully cope with their jobs. Misunderstandings regarding the disease can result in alienation and social isolation. Excessive care from caregivers further aggravates patients' feelings of guilt and negative thoughts.^11^, ^13^, ^14^, ^17^, ^18^, ^19^

The biggest burden is losing your job and not knowing [what to do next]. I had a permanent and secure employment. There are not enough positions for people that can't produce at the highest level. So, that's the problem [for the employer]; what to do about you?^18^

### 3.4 Self‐reorientation in the therapeutic process

In the treatment phase, patients with AF still experienced confusion and were not fully adapted to their health condition. However, at the same time, they attended moment to moment to their treatment and outcomes, and actively mobilized and utilized internal resources to maximize the therapeutic effect.

#### 3.4.1 Ambivalence in decision‐making

Variability existed in the extent to which physicians sought patients' opinions about treatment decisions and offered explanations of the treatment options. Patients' attitudes were mixed in terms of how much they wanted to be involved in decision‐making. Most were satisfied with the way the provider shared information to guide a treatment decision, while a few were unclear about the purpose of the received treatment and the anticipated risks.^12^, ^14^, ^16^, ^18^, ^19^, ^21^

Almost none of the patients could describe in their own words what AF is and why medical treatment was prescribed. They just took the medicine without knowing any details about how it would affect their disease because the cardiologist told them to.^12^

#### 3.4.2 Experiences during surgical interventions

During the relatively long procedures, conscious patients witnessed the surgical procedures and experienced physical and psychological discomfort. Emotions varied from expectation and fear to relief and a feeling of liberation at the end of the procedure. In addition, patients were required to face different types of physical suffering such as chest tightness, sweats and pain due to puncture and ablation. These feelings can encourage patients to actively mobilize their internal resources. During the administration of interventions and ablations, the technique of visualization was reported to significantly ameliorate anxiety, help in pain management and improve well‐being and satisfaction with treatment.^22^, ^23^

Well, I felt incredibly comfortable all the way in, so you could say that when she asked if I was nervous or anxious, I almost think I continually said no because I felt really comfortable.^23^

#### 3.4.3 Concerns and expectations regarding treatment

There can be a discrepancy between patients' expectations of treatment and the reality of treatment outcomes. Patients' expectations from early treatment were higher. They expected that the treatment would completely eliminate AF and that they would return to normal life posttreatment. However, only a minority of patients achieved longer‐term control of symptoms after treatment; most patients experienced unsatisfactory outcomes and feelings of mental defeat.^14^, ^15^, ^16^, ^18^

Patients were willing to try new treatment options after a particular treatment failed. Catheter ablation was usually the backup option among patients with failed direct‐current cardioversion. The properties of catheter ablation such as invasiveness and increased length of hospital stay increased the psychological burden on patients. It was worth noting that treatment efficacy was a key concern. Patients could reduce their expectations of retreatment concurrently to avoid disappointment. Some of the patients who experienced multiple unsuccessful treatment procedures, their expectations disappeared. As no further treatment options could be available, they often expressed negative attitudes.

Patients with AF were at an increased risk of stroke and thus required anticoagulant prophylaxis. Some patients expressed that accepting anticoagulation would bring a large hassle. This is relevant not only to an increased risk of bleeding after taking anticoagulants but also to the rigid requirements of monitoring warfarin therapy via the international normalized ratio. In recent years, novel oral anticoagulants have been applied to AF patients, and patient preferences varied for this new drug therapy. The formulation of anticoagulant therapy should be based on joint decisions made by the physicians and patients.^14^, ^15^, ^16^, ^17^, ^18^, ^19^, ^20^, ^21^

When you first go on warfarin you think flipping hell, I better not cut myself for brush my gums too hard.^16^

I cannot come to the hospital regularly for the blood tests. You know waiting in the line in front of that door is just killing me.^17^

### 3.5 Living with AF and QoL

As AF can be a chronic condition, it is important for patients to be able to live with this condition for an extended period of time. The ability to cope with this condition directly determines the QoL of patients and influences the rate of improvement in health.

#### 3.5.1 Cognitive coping strategies

Cognitive restructuring was the most frequently used coping strategy. Patients felt shock and resistance when they were initially diagnosed with AF; this went against consistent bodily perception. With disease progression and an increase in the severity of symptoms, patients were forced to accept the reality of their disease and to gradually establish a determination to fight against AF. At the same time, being labelled as an AF patient enhanced the value attached to health, and triggered them to think about life with rekindled hope for the future. People with AF strove to live in the present and to contribute to society. Many patients transitioned from having a negative attitude to thinking positively about life. Adapting to living with AF was a relatively favourable outcome second to obtaining a cure.^10^, ^12^, ^13^, ^15^, ^16^, ^17^, ^18^, ^19^

Because of this illness, I cannot eat or move the way I used to, but I really am thankful. There is always someone worse off than you. May God grant them a favour and patience! I think this is God's will. May God prevent a worse situation and help us protect our mental health!^17^

#### 3.5.2 Behavioural coping strategies

Patients also explored various behavioural coping strategies. They usually obtained disease‐ and treatment‐related information via multiple channels including the internet, by consulting with healthcare professionals and by sharing experiences with other patients. To avoid symptoms, patients actively monitored their physical states, identified triggers and actively worked to avoided them by slowing down or reducing specific activities. However, it is possible that in some cases, symptoms occurred when the patients were engaged in relaxation activities such as deep breathing. When these coping strategies were ineffective, patients expressed extremely negative emotions and adopted a negative coping style. However, cognitive restructuring was effective in improving patients' attitudes towards symptoms and their ability to cope with the disease.^11^, ^13^, ^15^, ^16^, ^17^, ^18^, ^19^

To break it—other things I did was cold water, ice cubes on my face—coughing real hard—deep breaths and coughing, but it seems in this calendar year, none of those things have worked.^15^

### 3.6 External support to facilitate coping strategies

Many patients experienced stress with regard to coping with the disease, and they expressed an urgent need for support in this regard. Receiving external support could help patients develop coping strategies and improve their QoL.

#### 3.6.1 Medical support

In most cases, regular follow‐up examinations were necessary to detect AF recurrence, monitor anticoagulant therapy and measure clinical outcomes. However, the cumbersome procedures of revisits consumed a lot of time and energy, especially with reference to monitoring anticoagulant therapy. Many patients have suggested opening outpatient AF clinics and simplifying the steps involved in the diagnosis and treatment of AF. The support of healthcare professionals was critical for AF patients. Appropriate communication behaviours during medical consultations could alleviate patient anxiety; some patients expressed a strong desire for a formal follow‐up schedule.^10^, ^12^, ^17^, ^19^, ^20^

Well, maybe there could be a separate unit where the blood test can be done and we could get the results.^17^

#### 3.6.2 Information support

The patient occupies a relatively weak position in a doctor–patient relationship, and thus needs enough support to close the information divide. In fact, most AF patients were not adequately knowledgeable about their condition. Despite significant health education, patients may not have understood the information adequately, or may have forgotten critical points. The internet is increasingly becoming one of the main resources for acquiring health information; however, the quality of information obtained by the patients was variable. Most people with AF had difficulties in processing complex information and expressed a need for proper education and easier modes of information delivery.^9^, ^10^, ^11^, ^12^, ^13^, ^14^, ^15^, ^16^, ^17^, ^19^, ^20^

But the doctor does not explain if they are beyond the coagulation or liquefaction limits. I just want doctors to talk to us more clearly and inform us.^17^

## 4 DISCUSSION

This review synthesized the perceptions of and experiences with AF in AF patients and revealed the complexity of the illness experience. Patients experienced long periods of uncertainty before obtaining a clear diagnosis of AF and reported feeling stress because of the gap between expectations of treatment and outcomes in real life. Moreover, patients underwent great changes with regard to physical, psychological, sociocultural and self‐worth aspects, and developed different coping strategies through experience. Many patients reported feeling uninformed and unsupported, and most studies recommended that effectively disseminating disease‐related information or services would help AF patients achieve better self‐management behaviours.

We used the COREQ checklist combined with the CASP tool to thoroughly review the quality and transparency of reporting of the included studies. The COREQ checklist provides a descriptive supplement for the criteria in CASP and proves the reliability of the quality grade. Using these two quality appraisal tools together could enhance the methodological rigour. More importantly, our findings were synthesized from different studies whose quality grades were strictly related to the credibility of the integrated results. Hence, we also performed a sensitivity analysis for the included studies and results. Interestingly, our findings are supported by studies with a quality grade of A or B; no conflicting viewpoints were presented in these studies. Therefore, we believe that our findings have high credibility.

Our results showed that patients experienced extended periods of uncertainty about the symptoms before AF was correctly diagnosed. This finding is concerning because a delayed or missed diagnosis increases the risk for stroke and heart failure, and this may in turn make AF more difficult to treat. Therefore, it is crucial to detect AF as early as possible. International initiatives advocate the implementation of screening for AF in clinical practice.^24^ Advances in wearable device technology will likely yield a variety of options for AF detection and AF burden assessment.^25^ Additionally, while many patients did not recognize the symptoms, some healthcare providers (especially primary physicians) also misinterpreted the symptoms and dismissed them as insignificant. It is necessary to strengthen the training of medical personnel with reference to knowledge and skills to ensure rapid identification of AF and to improve the AF diagnosis rate.^26^, ^27^

Therapeutic experiences were stage‐dependent, and varied in the pretreatment, ablation and posttreatment stages. First, the qualitative research included herein demonstrated recurring discordance between professionals and patients. Healthcare professionals reported shared decision‐making and patients experiencing a paternalistic model that is clinician‐dominated^28^; the healthcare professionals mistakenly felt that most patients preferred to defer decision‐making to their physician rather than participate in the process.^29^ Instead, our findings showed that many patients were eager to participate in treatment decisions, but did not do so due to knowledge gaps. To optimize shared decision‐making about AF treatment options, it is recommended that physicians should inform patients about the advantages/limitations and benefit/risks related to the treatment options and consider the patients' perception of potential treatment burden.^30^ Second, despite pharmacological analgesia during ablation, varying degrees of anxiety and discomfort were the most frequently reported problems. Hypnosis interventions have been proven to reduce the amount of pain medication used for patients undergoing minimally invasive procedures.^31^ At the same time, the presence of the medical staff was of great importance, as their solicitude and support likely provided a feeling of security for patients. Third, according to the Expectation Confirmation Theory, consumers' satisfaction depends on the extent to which consumer expectation meets perceived performance.^32^ Similarly, there was a decline in patient treatment satisfaction and trust because of the discrepancies between the expected and perceived effects. Informing patients about the limitations and risks of treatment methods combined with the implementation of lifestyle interventions can jointly improve clinical outcomes.

Our findings are in line with Lazarus and Folkman's^33^ stress‐coping model that has been used to explain how people cope with stressful events. The present study identified the presence of multiple burdens in patients across the trajectory of AF. Burden or stress is caused by a mismatch between the perceived demands of patients and the resources available to meet those demands. For example, several patients experienced emotional exhaustion due to the AF symptoms and their repeated recurrence; such patients tend to adopt evasive behaviours in AF‐related stress situations, which may further aggravate the stress reaction, leading to the generation of negative emotions such as depression and anxiety. If the patients are effectively motivated, the two psychological processes of emotional appraisal and coping strategies may greatly alleviate stress. The study findings indicated that most patients preferred to adopt positive appraisal methods (thinking, reasoning and decision‐making) and take positive action (seeking information and help). Beyond this, several studies^34^, ^35^, ^36^, ^37^ indicated that communal coping involving patients with a chronic disease and their spouses could not only promote mental and physical health of care recipients but also reduce the burden of care and improve the QoL of caregivers.

The presence of multiple support systems can reduce the AF‐related pressure load of patients and help improve mental health states.^38^ There was a significant need for medical, emotional and informational support among all participants in this study. At the hospital level, the construction of a telemedicine follow‐up management system is encouraged to improve efficiency in delivering healthcare. With the help of the telemedicine platform, remote experts can successfully complete long‐distance consultation, doctor–patient communication and impart information and knowledge. At the community level, various mutual‐help activities should be promoted to provide multiple emotion regulation channels. It is noteworthy that education settings and modes of information delivery affect the knowledge and understanding of AF information.^39^, ^40^ In this regard, using visual materials such as booklets along with video‐based animations as a substitute for oral advice and rebuilding a stronger social support system could increase the patients' level of knowledge about the disease and compliance with treatment; this is expected to enhance their positive coping levels.

### 4.1 Limitations

Our review has some limitations. First, even though nine databases were queried, it is possible that some eligible studies were missed. Second, we excluded studies only exploring the anticoagulation experience as it has been fully confirmed from the physicians' and patients' perspectives, respectively. Yet, it may have an influence on inferring reliable conclusions. Third, patients' experiences have been demonstrated to affect the spouses of the patients as well; however, this study did not include the family members of participants, and thus their preference was completely undetermined. In the future, it is vital to find effective ways to educate family members for improving the QoL in AF patients. Finally, two studies were not assessed using the COREQ checklist due to methodological limitations, which could weaken the overall assessment of confidence in the findings.

## 5 CONCLUSION

Our findings highlight the unique experiences of patients with AF. Delays in seeking medicine attention and delayed diagnosis make it difficult for patients to gain a deeper understanding of AF and its symptoms. Despite obtaining a definitive diagnosis of AF, patients were caught in a dilemma during the three‐stage treatment period. Specifically, they experienced confusion in decision‐making, anxiety and pain during surgical treatment, and disappointment or despair during recurrent episodes of AF and when unpredictable symptoms occurred. AF also adversely affected the physical, mental, social life and self‐worth aspects of patients. When faced with these challenges, patients were observed to aggressively attempt to establish a new life; they also expressed emotional needs and the need for further education. Future research and clinical practice are expected to improve the quality of medical diagnosis and treatment, optimize administrative strategies and provide diverse health support for patients with AF.

## CONFLICT OF INTERESTS

The authors declare that there are no conflict of interests.

## AUTHOR CONTRIBUTIONS

Wang Jie, Liu Shenxinyu, Sun Guozhen, Bao Zhipeng, Gao Min, Peng Yuanyuan, Wang Lin, Yu Tianxi and Huang Yangxi contributed to study design. Wang Jie, Liu Shenxinyu, Bao Zhipeng, Peng Yuanyuan and Gao Min contributed to data collection and data analysis. Wang Jie and Liu Shenxinyu contributed to writing of the manuscript. All authors contributed to manuscript revision and approval of the final submission.

## Data Availability

Data sharing is not applicable to this article as no data sets were generated or analysed during the current study.
